# Etiology of Chronic Renal Failure in Hodeida, Yemen

**DOI:** 10.1016/j.ekir.2025.103738

**Published:** 2025-12-20

**Authors:** Nabeel Yahya Al-madwahi, Ahmed Hasan Batah, Muneer Gamil Alwesabi, Haitham Mohammed Jowah

**Affiliations:** 1 Department of Surgery, Faculty of Medicine and Health Sciences, Sana’a University, Sana’a, Yemen; 2 Department of Vascular Surgery, Al-Thawra Modern General Hospital, Hodeida City, Yemen

To the Editor:

The etiology of chronic kidney disease (CKD) in Yemen remains understudied, particularly in Hodeida, a conflict-affected coastal region burdened by limited health care infrastructure and a high infectious disease burden. Although global patterns are dominated by diabetes and hypertension,^1^^,^^2^ we hypothesized that local environmental and socioeconomic factors might drive a different etiological profile in our region.

We conducted a cross-sectional study of 272 hemodialysis patients at the Al-Sammad Medical Center, Hodeida (Supplementary Methods). Our findings revealed a striking etiological profile: unknown causes were dominant in 61.8% (*n* = 168), followed by obstructive or infectious uropathy in 22.4% (*n* = 61) (Table 1). This high prevalence of unknown etiology largely reflects the severe diagnostic limitations in our setting, including the unavailability of kidney biopsy, immunofluorescence, and genetic testing, precluding the confirmation of specific diagnoses, such as glomerulonephritis or interstitial nephritis. Traditional causes were markedly less prevalent, including hypertensive nephropathy (8.8%) and diabetic nephropathy (2.9%).

**Table 1:** Etiological distribution of chronic renal failure (*N* = 272)

Primary etiology	Count (*n*)	Percentage (%)
Unknown etiology	168	61.8
Obstructive/Infectious uropathy	61	22.4
Hypertensive nephropathy	24	8.8
Diabetic nephropathy	8	2.9
Inherited/Congenital	6	2.2
Other specified causes	3	1.1
Glomerulonephritis	2	0.7
Total	272	100.0

The mean patient age was 42.9 ± 13.1 years, indicating that CKD predominantly affects Yemen’s economically active population (Supplementary Tables S1 and S2). Although hypertension was present in 79.4% of the cohort, it was identified as the primary etiology in only 8.8%. This discrepancy suggests that in the absence of histological confirmation, clinicians frequently classify hypertension as a secondary complication of CKD rather than the primary cause, potentially leading to underdiagnosis of hypertensive nephropathy in medical records.

Our findings mirror other hospital-based studies in Yemen, where undetermined etiologies consistently predominate.^3^ Although this pattern may partly align with CKD of unknown etiology described in agricultural communities globally,^4^ it primarily underscores the urgent need for improved diagnostic capacity. The predominance of obstructive uropathy further reflects inadequate early urological care, and the high prevalence of renal stones in Yemen’s coastal regions.^3^

These findings suggest a paradigm shift in regional CKD prevention strategies. Public health efforts should prioritize early urological intervention, investigation of environmental CKD drivers, and crucially, enhanced diagnostic capacity rather than solely focusing on diabetes and hypertension management, typical of high-income settings.

## Disclosure

All the authors declared no competing interests.

## Supplementary Material

Supplementary File (PDF) **Supplementary Methods. Table S1.** Baseline demographic and clinical characteristics of the study cohort (*N* = 272). **Table S2.** Comparative analysis of patients with unknown versus known CRF Etiology.

## Footnotes

Supplementary File (PDF)

**Supplementary Methods.**
**Table S1.** Baseline demographic and clinical characteristics of the study cohort (*N* = 272). **Table S2.** Comparative analysis of patients with unknown versus known CRF etiology.
