# Supplementary material for: Etiology of Chronic Renal Failure in Hodeida, Yemen
Source: Kidney Int Rep. 2025 Dec 20;11(3):103738. doi: 10.1016/j.ekir.2025.103738 (PMC12818221; doi:10.1016/j.ekir.2025.103738)
Supplement: Supplementary File (PDF) — Supplementary Methods. Table S1. Baseline demographic and clinical characteristics of the study cohort (N = 272). Table S2. Comparative analysis of patients with unknown versus known CRF Etiology. [file mmc1.pdf]

Supplementary Information for KIR-11-25-1982 Etiology of Chronic Renal Failure in Hodeida, Yemen

Supplementary Methods

Study Design and Setting

This study employed a cross-sectional analytical design to investigate the etiology and characteristics of chronic renal failure (CRF) in patients undergoing maintenance hemodialysis. This study was conducted at the Martyr Al-Samad Dialysis Center in Hodeida City, Yemen, which serves as a primary referral center for nephrology care in the region. Data were collected from May 2025 to July 2025.

Participants

The study population consisted of all adult patients (aged ≥ 15 years) with a confirmed diagnosis of end-stage renal disease (ESRD) who were undergoing regular hemodialysis at the study center during the data collection period. Of the 279 patients initially screened, 272 met the eligibility criteria and were included in the final analysis. Patients without a definitive diagnosis of CRF and those who declined to participate were excluded.

Variables

The primary outcome variable was CRF etiology. Secondary variables included patient demographics (age and sex), clinical characteristics (duration of diagnosis), major comorbidities (hypertension and diabetes mellitus), lifestyle factors (salt intake and exercise), and clinical complications (stroke, heart failure, and diabetic neuropathy).

Age was analyzed as both a continuous and categorical variable (<40, 40–59, and ≥60 years). To ensure a standardized classification of CRF causes, a primary etiological category was assigned to each patient based on their medical records and physician-reported diagnoses using a predefined clinical hierarchy as follows: (1) Diabetic Nephropathy; (2) Hypertensive Nephropathy; (3) Obstructive/Infectious Uropathy (a composite category including kidney stones, recurrent UTIs, and obstructive conditions); (4)

glomerulonephritis; (5) hereditary/congenital disorders; (6) Other Specified Causes; and (7) Unknown Etiology for all remaining cases where the cause was not specified or explicitly documented as unknown. This classification system is limited by the resource-constrained setting, and the assignment largely reflects the recorded clinical judgment based on available diagnostic tests, which were highly constrained.

Data Sources and Measurement

Data were collected through a combination of patient interviews using a structured questionnaire and a review of existing medical records. Trained healthcare personnel administered the questionnaires to ensure consistency and accuracy. It was designed to capture information on demographics, self-reported medical history, family history, lifestyle habits, and symptoms related to kidney disease and its complications. Clinical data, including the physician-diagnosed cause of CRF and the presence of comorbidities, were cross-validated with patient medical records where available.

Bias

To minimize selection bias, all eligible patients undergoing hemodialysis during the study period were invited to participate. To address potential information bias, particularly recall bias in self-reported data, interviewers used standardized prompts, and key clinical variables (e.g., etiology and comorbidities) were verified against medical records. However, the large proportion of "unknown etiology" may reflect diagnostic limitations in the clinical setting, which is a potential source of misclassification bias.

Study Size

The study size was determined by the total number of eligible and consenting patients at the participating center during the study period, resulting in a cohort of 272 individuals. The sample size was deemed sufficient to describe the primary etiological patterns within this specific clinical population.

Statistical Methods

All statistical analyses were performed using SPSS version 26.0. The primary analysis was purely descriptive. Descriptive statistics were used to summarize the data, and continuous variables are

presented as mean ± standard deviation (SD), whereas categorical variables are presented as frequencies and percentages (n, %). Missing data were handled by exclusion from specific analyses when data points were required.

Ethical Considerations

The study protocol was reviewed and approved by the Institutional Review Board of Al-Thawra Hospital, Hodeida City, Yemen. Written informed consent was obtained from all adult participants prior to their inclusion in the study. For participants under the age of 18 years, assent was obtained along with consent from a parent or legal guardian. All data were anonymized to ensure patient confidentiality.

Supplementary Table S1: Baseline Demographic and Clinical Characteristics of the Study Cohort (N=272)

**Characteristic**	**Value**
**Age**	
Mean (SD), years	42.9 (± 13.1)
Median (Range), years	42.0 (15 - 85)
**Gender, n (%)**	
Male	167 (61.4%)
Female	105 (38.6%)
**Age Group, n (%)**	
<40 years	112 (41.2%)
40-59 years	118 (43.4%)
≥60 years	42 (15.4%)
**Major Comorbidities, n (%)**	
Hypertension	216 (79.4%)
Diabetes Mellitus	34 (12.5%)
**Blood Pressure Control (among all patients), n (%)**	
Good Control	101 (37.1%)
Poor Control	92 (33.8%)
Unknown	79 (29.0%)
**Family History, n (%)**	
Family history of kidney disease	114 (41.9%)

SD: Standard Deviation

Supplementary Table S2: Comparative Analysis of Patients with Unknown versus Known CRF Etiology

Characteristic	Unknown Etiology (n=168)	Known Etiology (n=104)
**Mean Age (SD), years**	42.1 (± 13.6)	44.3 (± 12.1)
**Male Gender, n (%)**	100 (59.5%)	67 (64.4%)
**Hypertension, n (%)**	125 (74.4%)	91 (87.5%)
**Diabetes Mellitus, n (%)**	18 (10.7%)	16 (15.4%)

CRF, Chronic Renal Failure; SD: Standard Deviation.
